# Divalent magnesium cation concentrations determine the formation of tofu-like precipitates with differing urea solubilities

**DOI:** 10.1016/j.heliyon.2018.e00817

**Published:** 2018-09-24

**Authors:** Yasuhiro Arii, Kaho Nishizawa

**Affiliations:** a Department of Food Science and Nutrition, School of Human Environmental Sciences, Mukogawa Women's University, Nishinomiya, Hyogo 663-8558, Japan; b Department of Dietary Life and Food Sciences, Junior College Division, Mukogawa Women's University, Nishinomiya, Hyogo 663-8558, Japan; c Research Institute for Nutrition Sciences, Mukogawa Women's University, Nishinomiya, Hyogo 663-8558, Japan

**Keywords:** Analytical chemistry, Food technology, Food analysis, Food science

## Abstract

Different coagulant concentrations can induce urea-soluble precipitates (USPs) and urea-insoluble precipitates (UIPs) during tofu-like precipitate formation. In this study, MgCl_2_ concentration-dependent changes in USPs were quantified using a method based on urea solubility differences in order to investigate the factors affecting USP versus UIP formation. The addition of various Mg salts revealed that anions influence the solubility of proteins in both USPs and UIPs. Moreover, addition of MgCl_2_/NaCl mixtures, in which the Cl^−^ concentration was held constant, demonstrated that Mg^2+^ was essential for UIP formation but not for USP formation and that Cl^−^ was inconsequential for both USP and UIP formation. NaCl addition showed that the driving force for USP formation was salting-out due to the presence of cations. Overall, our data indicated that the Mg^2+^ concentration determined the separation of USPs and UIPs. These results will help elucidate the molecular mechanisms mediating the separation of silken tofu and regular tofu.

## 1 Introduction

Tofu can be classified as silken tofu and regular tofu, which are noticeably different in appearance and have different water contents (MEXT, 2015). The traditional process for making tofu involves adding a coagulant to soymilk and incubating the mixture at a high temperature (Watanabe and Saio, 1973). The soymilk is then separated into a precipitate and supernatant after the soy proteins have coagulated. Silken tofu can be made by changing the soy protein and coagulant concentrations used to make regular tofu (Watanabe and Saio, 1973). Although these processes have been studied for years, the mechanisms underlying the separation into silken tofu and regular tofu remain unclear at the molecular level.

Coagulant concentration is an important factor in traditional processes used to make tofu (Arii and Takenaka, 2013, 2014; Kao et al., 2003; Toda et al., 2003). Recently, developments in commercial processes have improved profits in the food industry, but have complicated our mechanistic understanding of tofu formation. Data from previous studies have shown that supernatant protein concentrations are dramatically decreased by the addition of CaCl_2_ (Arii and Takenaka, 2014; Kao et al., 2003; Toda et al., 2003) and MgCl_2_ (Arii and Takenaka, 2013). In our previous study, we reported that low or high concentrations of various coagulants induced the formation of tofu-like precipitates with a smooth (SP) or rough surface (RP), respectively (Arii and Takenaka, 2013, 2014), and clear solubility differences in 2 M urea and different water contents were observed for these precipitates (Arii and Takenaka, 2013). Based on these previous findings, we speculated that SP and RP may be relevant models for silken and regular tofu, respectively (Arii and Takenaka, 2013). SP and RP are mainly composed of urea-soluble precipitates (USPs) and urea-insoluble precipitates (UIPs), respectively (Arii and Takenaka, 2013). Therefore, studying USP and UIP formation may help elucidate the molecular mechanisms explaining the separation into silken and regular tofu during tofu preparation.

Accordingly, in the present study, we investigated and quantified MgCl_2_ concentration-dependent changes in USPs using a method based on differences in urea solubility. To determine the mechanisms mediating the separation into silken and regular tofu at the molecular level, the effects of anion concentrations on USP and UIP formation were investigated by adding different Mg salts. Using a constant Cl^−^ concentration, we analyzed USP and UIP formation by adding variable MgCl_2_/NaCl mixtures. These analyses revealed important factors affecting the formation of tofu-like precipitates with different physicochemical properties (i.e., SP and RP).

## 2 Materials and methods

### 2.1 Materials

Plain soymilk (40 mg protein/mL) was purchased from Nagoya Seiraku Co., Ltd. (Nagoya, Japan). Protein assay dye reagent concentrate was purchased from Bio-Rad Laboratories (Hercules, CA, USA). Other reagents were purchased from Wako Pure Chemicals (Osaka, Japan).

### 2.2 Preparation of various Mg solutions

Mg salts (MgCl_2_, MgSO_4_, MgBr_2_, Mg(NO_3_)_2_, and Mg(ClO_4_)_2_) and NaCl were dissolved in distilled water at various concentrations. In addition, MgCl_2_ was dissolved with NaCl in distilled water to prepare various MgCl_2_/NaCl mixtures containing different Mg^2+^ concentrations and constant Cl^−^ concentrations. In these mixtures, the Na^+^ concentration was decreased by increasing the Mg^2+^ concentration.

### 2.3 Preparation of tofu-like precipitates and supernatants from soymilk

Tofu-like precipitates and supernatants were prepared as described in our previous studies (Arii and Takenaka, 2013, 2014). Briefly, soymilk was incubated at 80 °C for 10 min (Watanabe and Saio, 1973; Toda et al., 2003). Coagulants were then dissolved in distilled water at the indicated concentrations. Coagulant solution (at various concentrations) was diluted 1:10 in the incubated soymilk and stirred well, and the resulting mixture was incubated at 80 °C for an additional 60 min (Watanabe and Saio, 1973; Toda et al., 2003). The mixture was incubated on ice for 15 min in an ice bath and then separated into supernatants and precipitates by centrifugation at 4,100 × *g* for 10 min at 4 °C. The concentration of residual proteins in the supernatant was determined as described below. The precipitate was used to determine the concentration of urea-soluble proteins, as described below.

### 2.4 Extraction of urea-soluble proteins

Urea-soluble proteins were extracted as described in our previous study (Arii and Takenaka, 2013), with slight modifications. The precipitate was suspended in 2 M urea in a volume 10-fold that of the initial soymilk, incubated at 50 °C for 60 min, and agitated every 15 min with a vortex mixer. After incubation, the sample was separated into a supernatant and precipitate by centrifugation at 4,100 × *g* for 10 min at 4 °C. The concentration of urea-soluble proteins in the supernatant was determined as described below.

### 2.5 Determination of supernatant protein concentrations

Supernatant proteins were quantified by the Bradford method with a protein assay dye reagent concentrate (Bio-Rad Laboratories). The ratio of the residual protein concentration was expressed as the protein concentration in the supernatant after coagulant addition to that of soymilk diluted 10:1 by distilled water. The ratio of the urea-soluble protein concentration was expressed as the concentration of urea-soluble proteins to the protein concentration of soymilk diluted 10:1 by distilled water. The data shown represent the average ± standard deviation of three independent experiments.

### 2.6 Normalization of urea-soluble protein concentration ratios

To compare changes in urea-soluble protein concentrations, the normalized ratio (*r*_n_) was calculated using the following equation:

(1) *r*_n_ = (*r* − *r*_min_) / (*r*_max_ − *r*_min_)

where *r*, *r*_min_, and *r*_max_ are the percentage of each urea-soluble protein concentration, the minimum percentage, and the maximum percentage, respectively, as determined by adding the Mg salt solution or MgCl_2_/NaCl mixture, as described above.

### 2.7 Statistical analysis

Student's *t*-tests were used to investigate the effects of NaCl addition on the quantity of urea-soluble proteins. Differences with *p* values of less than 0.05 were considered statically significant.

## 3 Results and discussion

### 3.1 Detection of the transition point from USP formation to UIP formation

**Fig. 1:**
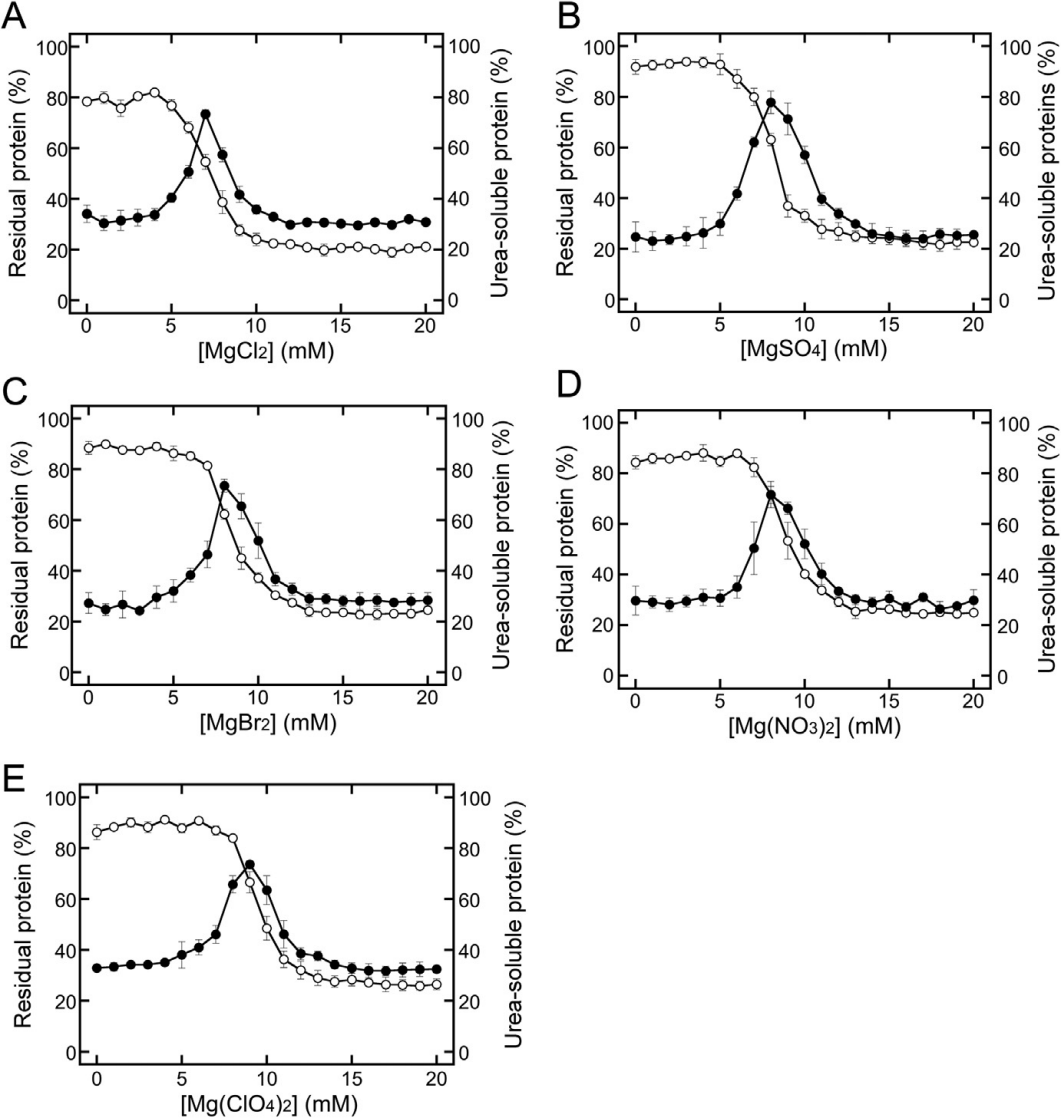
Changes in residual and urea-soluble proteins in the presence of various Mg salt solutions. MgCl_2_ (A), MgSO_4_ (B), MgBr_2_ (C), Mg(NO_3_)_2_ (D), and Mg(ClO_4_)_2_ (E) were added to soymilk at the indicated concentrations. Open and closed circles represent residual protein concentrations and urea-soluble protein concentrations, respectively. The data are shown as the averages ± standard deviations of three independent experiments.

In our previous study, we reported that tofu-like precipitates prepared with a low concentration of MgCl_2_ show higher solubility in 2 M urea than precipitates prepared with a high concentration of MgCl_2_ (Arii and Takenaka, 2013). A detailed analysis of this phenomenon would provide crucial chemical information for elucidating the mechanisms mediating the separation into SP and RP. To investigate and quantify the effects of MgCl_2_ concentration on changes in USPs, precipitates prepared by the addition of MgCl_2_ were suspended in 2 M urea. Soymilk was separated into supernatants and precipitates by incubation with MgCl_2_, followed by centrifugation, and the concentration of proteins remaining in the supernatant was measured (Fig. 1A). The residual protein concentration changed sigmoidally, similar to the results in our previous studies (Arii and Takenaka, 2013, 2014), and tofu-like precipitate formation was also observed. Urea-soluble proteins increased with increasing MgCl_2_ concentrations over the range of 0–7 mM and then decreased above 7 mM MgCl_2_ (Fig. 1A). Urea-soluble protein concentration reached a minimum at 12 mM MgCl_2_. The increase in urea-soluble proteins indicated that USP was increased in an MgCl_2_ concentration-dependent manner in the range of 0–7 mM. In contrast, the supernatant proteins and urea-soluble proteins were decreased in an MgCl_2_ concentration-dependent manner above 7 mM MgCl_2_. Thus, the decrease in supernatant proteins was associated with an increase in tofu-like precipitate (Arii and Takenaka, 2013, 2014). The decrease in urea-soluble proteins implied that UIPs were increased in an MgCl_2_ concentration-dependent manner above 7 mM MgCl_2_. In other words, the MgCl_2_ concentration of 7 mM represented the transition point from USP formation to UIP formation. Based on these findings, we established a method for detecting the transition point from USP formation to UIP formation.

### 3.2 Effects of different anions on USP and UIP formation

**Fig. 2:**
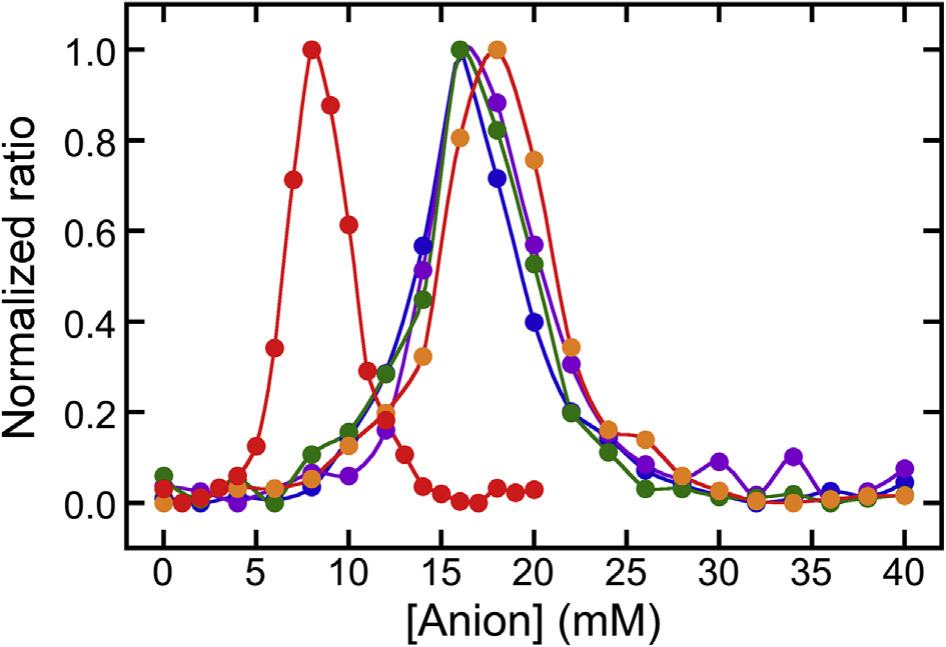
Comparison of the urea-soluble protein concentration observed with different Mg salts. The normalized ratios were calculated based on the concentrations of urea-soluble proteins shown in Fig. 1, using Eq. (1). The ratio was plotted against the anion concentration. The concentration of monovalent anions (Cl^−^, Br^−^, NO_3_^−^, and ClO_4_^−^) was calculated as double that of coagulants (MgCl_2_, blue circles and line; MgBr_2_, green circles and line; Mg(NO_3_)_2_, purple circles and line; and Mg(ClO_4_)_2_, orange circles and line, respectively). The MgSO_4_ concentration was equivalent to that of the divalent anion SO_4_^2−^ (red circles and line).

Different urea solubilities were used to investigate the effects of anions on USP and UIP formation. In addition to MgCl_2_ (Fig. 1A), other Mg salts, such as MgSO_4_ (Fig. 1B), MgBr_2_ (Fig. 1C), Mg(NO_3_)_2_ (Fig. 1D), and Mg(ClO_4_)_2_ (Fig. 1E), were added to soymilk. The Mg salts showed similar effects on the residual proteins and urea-soluble proteins, although some differences were noted (Fig. 1). The observed behaviors were similar, indicating that Mg salts other than MgCl_2_ could also induce USP and UIP formation. The behaviors with different anions were compared to investigate their roles in USP and UIP formation. The data for the urea-soluble proteins shown in Fig. 1 were normalized by Eq. (1) to compare the effects of different anions (at various concentrations) in more detail (Fig. 2). The peak concentrations approximated the order of the Hofmeister series (Broering and Bommarius, 2005; Hofmeister, 1888; Kunz et al., 2004; Zhang and Cremer, 2006): SO_4_^2−^ < Cl^−^ < Br^−^ ≈ NO_3_^−^ < ClO_4_^−^. As described in Fig. 1A, the peak concentrations implied a shift from USP formation to UIP formation. In addition, when the normalized ratio was 0.5, the anion concentration was defined as the midpoint concentration (*C*_m_). The *C*_m_ values for USP and UIP formation also approximated the order of the Hofmeister series (Broering and Bommarius, 2005; Hofmeister, 1888; Kunz et al., 2004; Zhang and Cremer, 2006): SO_4_^2−^ < Cl^−^ < Br^−^ ≈ NO_3_^−^ < ClO_4_^−^; these data indicated that identity of the anion influenced the protein stability during tofu-like precipitate formation.

The Hofmeister effect is still poorly understood (Wilson, 2012). However, anions are known to help proteins denature or dissolve and can also alter many other solution properties, such as surface tension (Zhang and Cremer, 2006). According to the Hofmeister effect, strong ion-specific destabilization typically occurs at salt concentrations higher than 0.5 M (Crevenna et al., 2012). The salt concentrations used for making tofu are much lower than 0.5 M. However, data from our previous study showed that SP proteins were less denatured than RP proteins (Arii and Takenaka, 2013), and even a low anion concentration can help destabilize soymilk proteins and encourage partial denaturation. These findings demonstrated that anions could partially denature proteins during tofu formation.

### 3.3 Protein solubility in the presence of various fixed Cl^−^ concentrations

**Fig. 3:**
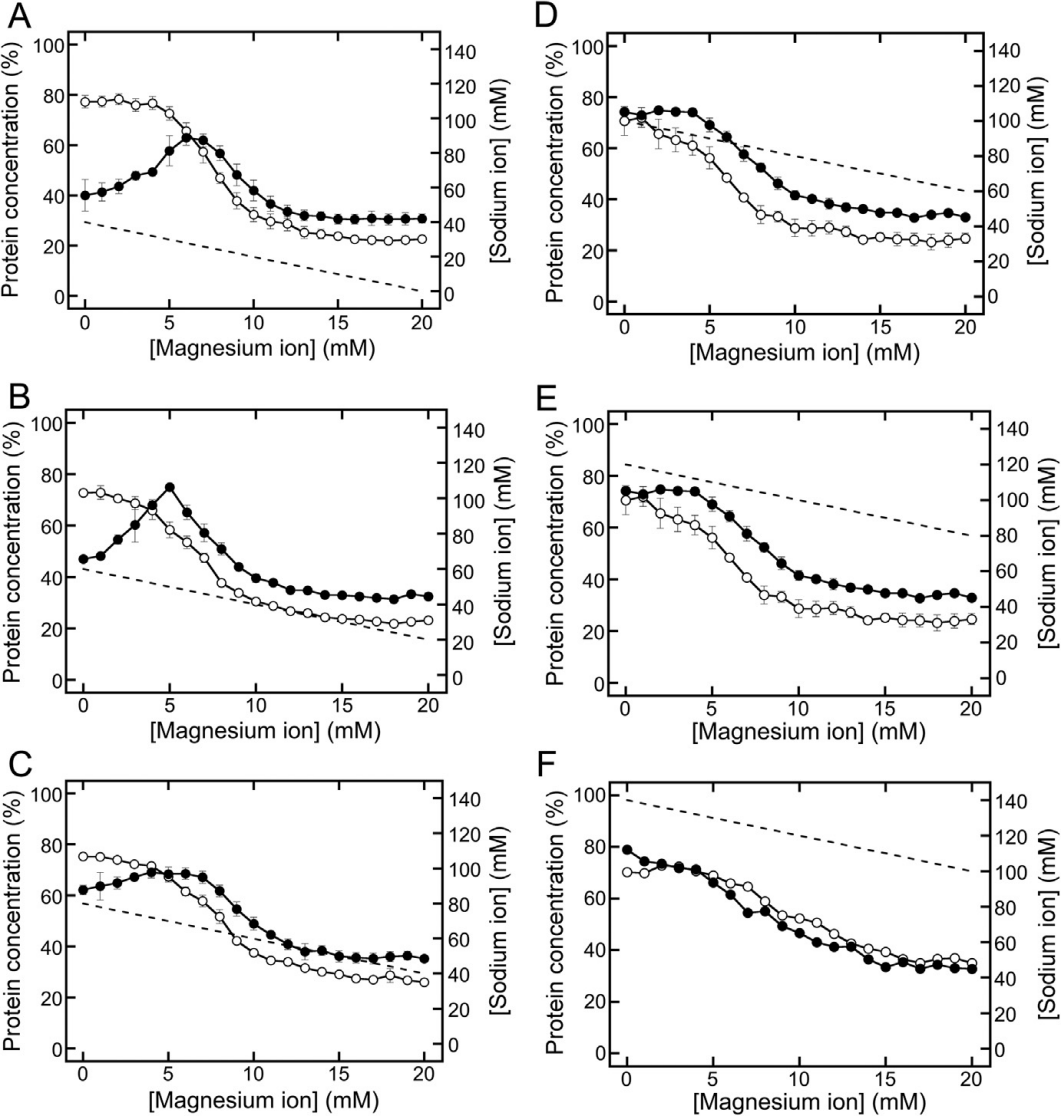
Changes in urea-soluble proteins prepared in the presence of various fixed Cl^−^ concentrations. MgCl_2_/NaCl mixtures were added to soymilk with various cation concentrations and various fixed Cl^−^ concentrations. The final Mg^2+^ concentration was in the range of 0 mM–20 mM in the presence of chloride ions at a constant concentration of 40 (A), 60 (B), 80 (C), 100 (D), 120 (E), or 140 mM (F). Open and closed circles represent residual protein concentrations and urea-soluble protein concentrations, respectively. The dashed lines represent the Na^+^ concentration. The data are shown as the averages ± standard deviations of three independent experiments.

Under various fixed Cl^−^ concentrations, quantitative changes in USPs were observed after changing the Mg^2+^ and Na^+^ concentrations (Fig. 3). MgCl_2_/NaCl mixtures were prepared with fixed Cl^−^ concentrations and were added separately to soymilk samples at final Cl^−^ concentrations ranging from 40 to 140 mM. While preparing the mixtures, when the Mg^2+^ concentration was increased, the Na^+^ concentration was decreased accordingly (Fig. 3). The concentration of urea-soluble proteins changed more dramatically after increasing the Cl^−^ concentration (Fig. 3). When the Cl^−^ concentration was increased by 40 (Fig. 3A), 60 (Fig. 3B), and 80 mM (Fig. 3C), the peak protein concentrations were observed at lower Mg^2+^ concentrations (6, 5, and 4 mM Mg^2+^, respectively), in contrast with the peak protein concentration observed at 7 mM Mg^2+^ in the absence of NaCl (Fig. 1A). At higher Cl^−^ concentrations (100 and 120 mM), the concentration of urea-soluble proteins decreased sigmoidally with increasing Mg^2+^ concentrations (Fig. 3D and E). Interestingly, in the presence of 140 mM Cl^−^, the concentration of urea-soluble proteins decreased in an almost linear manner (Fig. 3F). Fig. 3 shows the possibility that protein solubilities were influenced by the Cl^−^, Mg^2+^, and Na^+^ concentrations. In addition, the NaCl concentration was found to have a clear effect on USP formation during tofu formation (Fig. 3). As described in a previous report (Nagano and Tokita, 2011), a low ionic strength accelerates the rate of formation of crosslinked proteins by Mg^2+^ addition to induce a nonuniform, rough gel. In contrast, a high ionic strength deaccelerates the rate of formation of crosslinked proteins to induce a uniform gel (Nagano and Tokita, 2011). Thus, the dramatic changes in NaCl concentrations would also influence precipitate formation of soymilk proteins and could affect the appearance of precipitates. As shown in Table 1, tofu is produced commercially by adding a mixture of NaCl and MgCl_2_ (as a coagulant) to soymilk (Haga et al., 2005; Soeda and Yamazaki, 2002, 2003, 2004). These results implied that the addition of NaCl may make tofu production easier.

**Table 1:** Comparison of sodium, magnesium and calcium contents.

	Na (mg/100 g food)	Mg (mg/100 g food)	Ca (mg/100 g food)
Soymilk	2	25	15
Regular tofu	59	130	86
Silken tofu	14	55	57
Okinawa tofu	170	66	66

Data were cited from Standard Tables of Food Composition in Japan 2015 (Seventh Edition) (MEXT, 2015).

### 3.4 Comparison of USP and UIP formation with variable fixed Cl^−^ concentrations

**Fig. 4:**
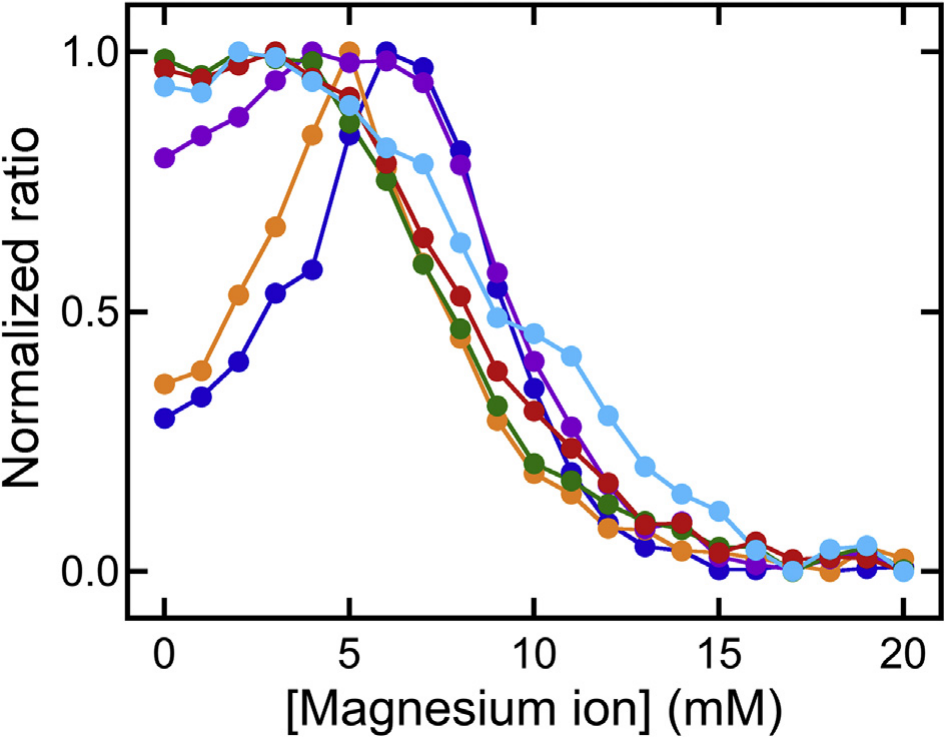
Comparison of the urea-soluble protein concentrations at different Cl^−^ concentrations. (A) The normalized ratio was calculated from the concentration of urea-soluble proteins shown in Fig. 3 using Eq. (1). The ratio was plotted against the Mg^2+^ concentration in the presence of Cl^−^. The Cl^−^ concentration was 40 (blue circles and line), 60 (orange circles and line), 80 (purple circles and line), 100 (green circles and line), 120 (red circles and line), or 140 mM (cyan circles and line).

The effects of the Cl^−^ concentration on USP and UIP formation were investigated by comparing changes in urea-soluble protein levels with differing fixed Cl^−^ concentrations (Fig. 4). The data for urea-soluble proteins shown in Fig. 3 were normalized using Eq. (1) and plotted versus the Mg^2+^ concentration (Fig. 4). With lower Mg^2+^ concentrations (<3 mM), the urea solubility of proteins increased in parallel with increasing Cl^−^ concentrations ranging from 40 to 100 mM (Fig. 4). The Cl^−^ concentration-dependent increase in urea-soluble proteins revealed the possibility that the Cl^−^ concentration may be important for USP formation. However, the Na^+^ concentration also increased with increasing Cl^−^ concentrations (Fig. 4), indicating the possibility that the Na^+^ concentration may be important for USP formation. Additional studies are needed to further characterize the relative importance of Na^+^ and Cl^−^ in USP formation.

At higher Mg^2+^ concentrations (>5 mM), UIP formation was independent of the Cl^−^ concentration (Fig. 4). This Cl^−^ concentration-independence demonstrated that the Cl^−^ concentration was not critical for UIP formation. However, as shown in Fig. 2, the anion concentration influenced protein stability and solubility during tofu-like precipitate formation. In addition, UIP formation was not enhanced by adding only NaCl at a high concentration in the absence of MgCl_2_ (Fig. 4). This observation demonstrated that Na^+^ could not promote UIP formation in the range of NaCl concentrations tested. Taken together, our results showed that the Mg^2+^ concentration was a critical factor for UIP formation.

### 3.5 Role of NaCl in USP and UIP formation

To investigate the role of NaCl in USP and UIP formation in more detail, we focused on the levels of residual proteins (open circles of Figs. 1A and 3) and urea-soluble proteins (closed circles of Figs. 1A and 3) in the absence of Mg^2+^ (0 mM Mg^2+^ in Figs. 1A and 3) and in the presence of sufficient Mg^2+^ (20 mM Mg^2+^ in Fig. 3) at different Cl^−^ concentrations. In the absence of Mg^2+^, the amount of residual proteins were 78.5 ± 5.8 % at 0 mM Cl^−^ (Fig. 1A), 77.2 ± 2.5 % at 40 mM Cl^−^ (Fig. 3A), 72.7 ± 1.1 % at 60 mM Cl^−^ (Fig. 3B), 75.3 ± 1.1 % at 80 mM Cl^−^ (Fig. 3C), 70.6 ± 5.6 % at 100 mM Cl^−^ (Fig. 3D), 67.7 ± 3.1 % at 120 mM Cl^−^ (Fig. 3E), and 79.0 ± 2.0 % at 140 mM Cl^−^ (Fig. 3F). The concentration of the urea-soluble proteins (closed circles of Figs. 1A and 3) were shown to be 34.1 ± 4.8 % at 0 mM Cl^−^ (Fig. 1A), 40.0 ± 6.3 % at 40 mM Cl^−^ (Fig. 3A), 47.0 ± 1.3 % at 60 mM Cl^−^ (Fig. 3B), 62.2 ± 1.8 % at 80 mM Cl^−^ (Fig. 3C), 74.2 ± 0.3 % at 100 mM Cl^−^ (Fig. 3D), 74.4 ± 2.3 % at 120 mM Cl^−^ (Fig. 3E), and 70.3 ± 5.9 % at 140 mM Cl^−^ (Fig. 3F). Briefly, the amount of residual proteins showed almost no change with the increase in the NaCl concentration, whereas the urea-soluble proteins increased at NaCl concentrations over 60 mM and plateaued at an NaCl concentration of 100 mM. This increase indicated that NaCl promoted maximum USP formation at concentrations of 100 mM or more. Takahashi et al. (1982) reported that the viscosity of soy proteins markedly increased in the presence of Cl^−^ concentrations ranging from 100 to 200 mM, resulting in gel formation and a smooth surface. Similarly, our results also indicated that USPs with a smooth surface were markedly increased in the presence of Cl^−^ concentrations of more than 100 mM. Thus, the addition of NaCl at a high concentration promoted USP formation, although it is unclear whether USP was induced equally well by adding MgCl_2_. In addition, it is also unclear whether USP formation was promoted by adding Na^+^ or Cl^−^. In contrast, in the presence of sufficient Mg^2+^ (20 mM of Mg^2+^, Fig. 3) to induce UIP formation, both residual and urea-soluble protein levels were unchanged by increasing the NaCl concentration. These results indicated that the NaCl concentration was not an essential factor in UIP formation.

### 3.6 Roles of cations and anions in USP and UIP formation

As shown in Fig. 1A, the maximum concentration of urea-soluble proteins in the absence of NaCl was 73.4 ± 1.8 % at 7 mM MgCl_2_ (14 mM Cl^−^). In addition, as described earlier, in the absence of MgCl_2_, the concentration of urea-soluble proteins was 74.2 ± 0.3 % at 100 mM NaCl, 74.4 ± 2.3 % at 120 mM NaCl, and 70.3 ± 5.9 % at 140 mM NaCl. These data show that in case of MgCl_2_ addition as well as in the case of NaCl addition, the maximum concentration of urea-soluble proteins were nearly equal (approximately 75%), but the Cl^−^ concentration was vastly different when either MgCl_2_ (14 mM Cl^−^) or NaCl (>100 mM Cl^−^) were added. With NaCl addition, a finer resolution of the Cl^−^ concentration (every 2 mM Cl^−^) was also analyzed in the range of 0–40 mM of Cl^−^ (Data not shown). These results also indicated that NaCl addition clearly produced no USPs in the range of 0–40 mM Cl^−^. If the Cl^−^ concentration is an essential factor for USP formation, then USP formation should occur when only NaCl is added at a concentration of 14 mM. However, no USP formation occurred at this Cl^−^ concentration (14 mM) with only NaCl addition (Data not shown). Our results also demonstrated that the anion concentration was an inconsequential factor for USP formation. As described in Figs. 1 and 2, anions partially denatured proteins during tofu formation. From these findings, we concluded that the anion caused partial changes in protein structure, but was not a crucial factor for USP formation.

Moreover, from the data of urea-soluble proteins described above, it was noted that UIP formation was induced by adding only Mg^2+^ (but not Na^+^), suggesting that Mg^2+^ addition was essential for UIP formation. In contrast, USP formation was induced by adding only Mg^2+^ or only Na^+^. Based on these differences, we assumed that salting-out of soy proteins occurred during USP formation by cation interactions with the surface carboxyl groups of soy proteins and that the linkage between soy proteins through salt bridges was an essential factor for UIP formation. Cations would have different functions during separation into USPs and UIPs during tofu-like precipitate formation. Interestingly, Xu et al. (2015) reported that the aggregative behavior of soy proteins depended on the NaCl concentration, with signs of sedimentation observed at a concentration of 100 mM but dispersion occurring again at 400 mM. The concentration for the sedimentation was consistent with the concentration needed for USP induction by NaCl. Additionally, they also reported two competing effects of NaCl: salting-out and salting-in; these effects were attributed to complex electrostatic interactions between the soy proteins (Xu et al., 2015). As shown in Figs. 1 and 2, anion concentration influenced the partial denaturation of soymilk proteins. However, the anion concentration was not a crucial factor for separation into USPs and UIPs, but rather affected the urea solubilities of USPs and UIPs.

In the Japanese industrial manufacturing of tofu, the concentration of soy proteins in soymilk used to make silken tofu is higher than that used to make regular tofu (Nito, 2004). A higher density of proteins could be aggregated more easily by promoting weak interactions during salting-out. Interestingly, adding CaCl_2_ and CaSO_4_ at concentrations of approximately 10 mM during the preparation of silken tofu facilitated a suitable protein yield and texture (Beddows and Wong, 1987; Hashizume and Ka, 1978). At that concentration, USPs stopped decreasing during tofu-like precipitate formation (Fig. 1A), and the concentration was almost the same as the *C*_m_ value for RP formation by adding MgCl_2_ or CaCl_2_ reported in our previous study (Arii and Takenaka, 2014). This concentration could be favorable for making silken tofu based on a balance between salting-out and linkage through salt bridging.

In our previous study, USPs and UIPs were detected in tofu-like precipitates with SP and RP, respectively, by suspension in 2 M urea (Arii and Takenaka, 2013). This concentration (2 M) was suitable for detection of the separation of SP and RP. It is possible that the concentration of urea may be important for breaking some molecular interactions of USPs but not breaking the molecular interactions of UIPs. Separation of SP and RP, which resemble silken and regular tofu, respectively, is achieved by adding MgCl_2_ at different concentrations (Arii and Takenaka, 2013). Moreover, the relationship between the water contents of SP and RP was similar to the general relationship between silken and regular tofu (Arii and Takenaka, 2013). We speculate that SP and RP are relevant respective models for silken tofu and regular tofu. However, since SP and RP were loosely defined terms, it was difficult to analyze these in more detail. Our current findings indicated that separation by adding MgCl_2_ at different concentrations could be detected by suspension in 2 M urea, although USPs and UIPs may not be completely equivalent to SP and RP, respectively. This concentration (2 M) was most suitable for detection of the separation of SP and RP, suggesting that the concentration of urea may be useful for breaking the molecular interactions of USPs, but not breaking the molecular interactions of UIPs. Thus, studying USP and UIP formation may help elucidate the molecular mechanisms relevant to tofu preparation.

### 3.7 Interpretation of the excess sum of detected proteins

**Fig. 5:**
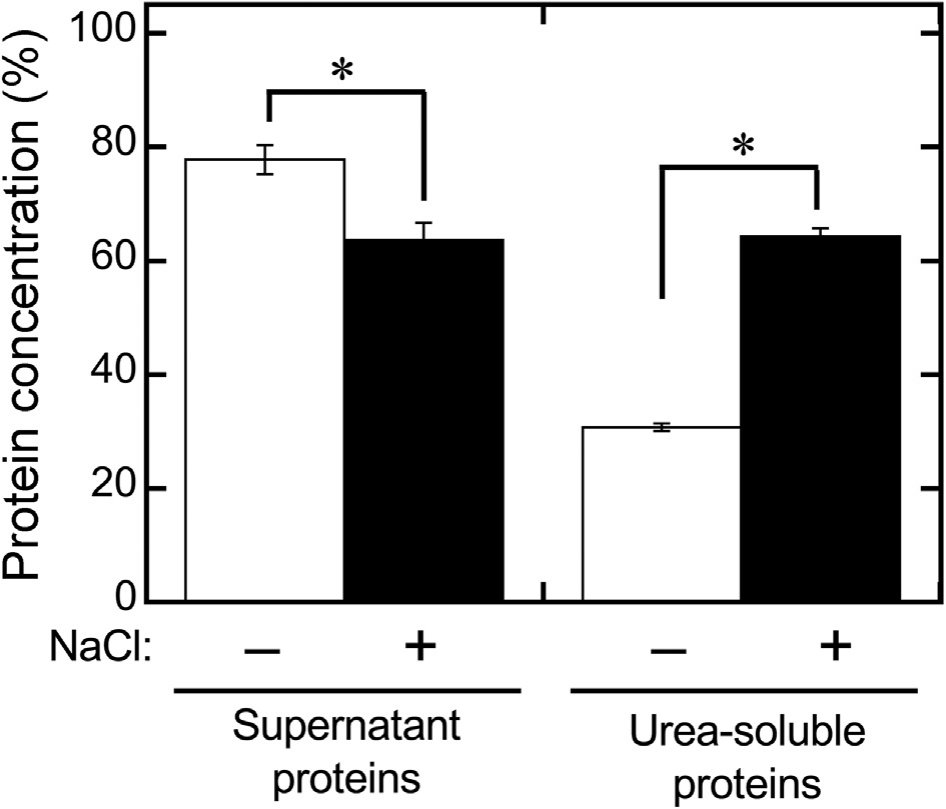
Changes in the total amount of detectable soy proteins observed by adding NaCl. Distilled (–) water or 1 M NaCl (+) were added to soymilk. The final NaCl concentration was 100 mM. The mixture was separated into supernatants (white boxes) and precipitates by centrifugation. Precipitates were suspended in 2 M urea (black boxes), and protein concentrations were determined by the Bradford method. The ratio was expressed as the protein concentration of the samples to that in initial soymilk prepared by adding distilled water without centrifugation. The data shown represent the averages ± standard deviations of three independent experiments. The statistical significance of differences was determined by Student's *t*-tests. *p < 0.05.

The sum of the residual proteins and urea-soluble proteins exceeded 100% at the same MgCl_2_ concentration (Figs. 1 and 3). This excess was induced by adding NaCl and dissolving precipitates with urea to increase the quantity of proteins detectable by the Bradford method (Fig. 5). In soymilk, many proteins interact with oil bodies (Chen and Ono, 2010; Ono, 2017). A fraction of proteins is undetectable by the Bradford method, including soy proteins in colloidal particles and soy proteins in oil-in-water emulsions with diminished protein surface charges. NaCl and urea addition can release such sequestered proteins, thereby increasing their detectability. In addition, the detectability would be increased with the increase in USPs because salting-out of USPs is easy to achieve with urea addition.

**Fig. 6:**
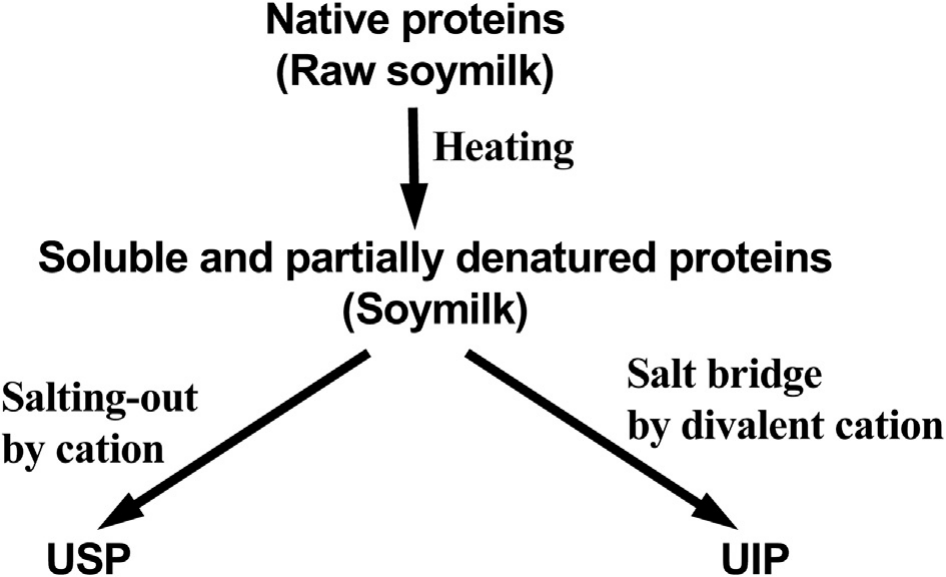
Schematic depiction of the formation of USPs and UIPs. The arrows from native proteins to soluble and partially denatured proteins indicate the production of soymilk from raw soymilk by heating at 100 °C, according to the models from Kohyama et al., 1995, Ono et al., 1991; and Shun-Tang et al. (1997).

## 4 Conclusion

In this study, we found that both Mg^2+^ and Na^+^ induced USPs, strongly suggesting that the driving force for USP formation was salting-out due to the interactions of cations with the surface carboxyl groups of soy proteins. Mg^2+^ was essential as an initiation factor for UIP formation. Anions were found to be inconsequential for USP and UIP formation but clearly influenced the degree of protein denaturation. The different urea solubilities observed between USPs and UIPs may have been induced by different anion concentrations. Based on these data, we concluded that the different roles of cations determined the outcome of USP and UIP formation (Fig. 6). These mechanistic data may help determine factors crucial for separation into silken and regular tofu.

## Declarations

### Author contribution statement

Yasuhiro Arii: Conceived and designed the experiments; Performed the experiments; Analyzed and interpreted the data; Contributed reagents, materials, analysis tools or data; Wrote the paper.

Kaho Nishizawa: Performed the experiments; Analyzed and interpreted the data; Contributed reagents, materials, analysis tools or data.

### Funding statement

This work was supported by the Tojuro Iijima Foundation for Food Science and Technology under Grant Number 2016-37.

### Competing interest statement

The authors declare no conflict of interest.

### Additional information

No additional information is available for this paper.
